# Evaluation of a Large Language Model’s Ability to Assist in an Orthopedic Hand Clinic

**DOI:** 10.1177/15589447241257643

**Published:** 2024-06-22

**Authors:** Travis Kotzur, Aaron Singh, John Parker, Blaire Peterson, Brian Sager, Ryan Rose, Fred Corley, Christina Brady

**Affiliations:** 1 UT Health San Antonio, TX, USA

**Keywords:** artificial intelligence, ChatGPT, GPT-4, large language model, machine learning, hand surgery, orthopedics

## Abstract

**Background:**

Advancements in artificial intelligence technology, such as OpenAI’s large language model, ChatGPT, could transform medicine through applications in a clinical setting. This study aimed to assess the utility of ChatGPT as a clinical assistant in an orthopedic hand clinic.

**Methods:**

Nine clinical vignettes, describing various common and uncommon hand pathologies, were constructed and reviewed by 4 fellowship-trained orthopedic hand surgeons and an orthopedic resident. ChatGPT was given these vignettes and asked to generate a differential diagnosis, potential workup plan, and provide treatment options for its top differential. Responses were graded for accuracy and the overall utility scored on a 5-point Likert scale.

**Results:**

The diagnostic accuracy of ChatGPT was 7 out of 9 cases, indicating an overall accuracy rate of 78%. ChatGPT was less reliable with more complex pathologies and failed to identify an intentionally incorrect presentation. ChatGPT received a score of 3.8 ± 1.4 for correct diagnosis, 3.4 ± 1.4 for helpfulness in guiding patient management, 4.1 ± 1.0 for appropriate workup for the actual diagnosis, 4.3 ± 0.8 for an appropriate recommended treatment plan for the diagnosis, and 4.4 ± 0.8 for the helpfulness of treatment options in managing patients.

**Conclusion:**

ChatGPT was successful in diagnosing most of the conditions; however, the overall utility of its advice was variable. While it performed well in recommending treatments, it faced difficulties in providing appropriate diagnoses for uncommon pathologies. In addition, it failed to identify an obvious error in presenting pathology.

## Introduction

The emergence of ChatGPT, a large language model (LLM) developed by OpenAI, has sparked discussions across disciplines on potential applications in research, education, and clinical practice.^1^ ChatGPT is a generative pretrained transformer, meaning it is a neural network that has been trained on a vast quantity of text. It can answer a variety of queries, via a novel text response, in a human-like manner. While it has no specific medical training, it has access to massive quantities of data, allowing it to answer many scientific and clinical questions. Indeed, Kung et al^2^ reported that ChatGPT was able to pass the United States Medical Licensing Examination (USMLE) battery of examinations and suggested that LLMs have the potential to aid in clinical decision-making.

While these results are promising, there remains significant work before this technology can be safely and efficiently incorporated into a clinical setting. For example, Patel and Lam proposed the application of ChatGPT to discharge summaries and provided an example of an AI-generated summary; however, they did not assess this application in any rigorous or systematic way.^3^ Furthermore, not all proposed applications have been successful. Wagner and Ertl-Wagner found that, when prompted with questions pertaining to clinical radiology, a third of ChatGPT-generated responses were incorrect, and references provided were either incorrect or inauthentic.^4^ Still, Lee et al^1^ suggested the possibility for ChatGPT to provide “curbside consults,” noting its ability to synthesize large quantities of data, such as those in medical records. Finally, a recent publication by Seth et al^5^ demonstrated the ability of ChatGPT to provide general information regarding carpal tunnel syndrome to patients. They suggested that LLMs could potentially function as supportive tools for clinicians.

Similarly, multiple papers from salient literature, cited in our manuscript, suggest LLMs had the potential to aid in clinical tasks.^6-11^ Furthermore, an updated GPT, known as GPT-4, with more advanced capabilities was recently released.^12^ This study aims to assess GPT-4’s ability to diagnose conditions, suggest an appropriate workup, and provide treatment options to assist orthopedic surgeons in the clinical setting.

## Methods

Nine original clinical vignettes, representing various presentations of both common and uncommon hand pathology based on real patients, were prepared by an orthopedic surgery resident and an independently practicing fellowship-trained orthopedic hand surgeon. The conditions included trigger finger, Dupuytren’s contracture, sagittal band rupture, radial nerve palsy, lateral epicondylitis, De Quervain’s tenosynovitis, posterior interosseous nerve syndrome, swan neck deformity, and mallet finger. ChatGPT was presented with these vignettes and instructed to generate a differential diagnosis, create a workup plan, and expound on treatment options as if it were assisting a surgeon in an orthopedic hand clinic.

To ensure fair testing, ChatGPT was allowed to justify its reasoning if it failed to include the correct diagnosis in its differential. In addition, to test ChatGPT’s attention to detail, one of the diagnoses (radial nerve palsy) was presented with an incorrect nerve distribution.

**Figure 1.**
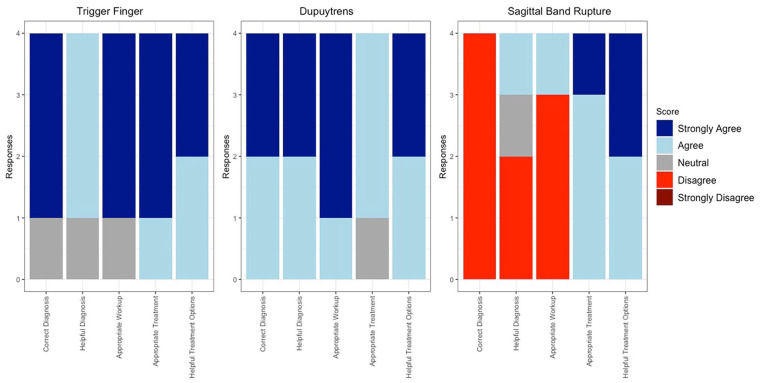
Stacked bar graph depicting individual surgeon responses to each vignette’s 1, 2, and 3 Likert survey.

**Figure 2.**
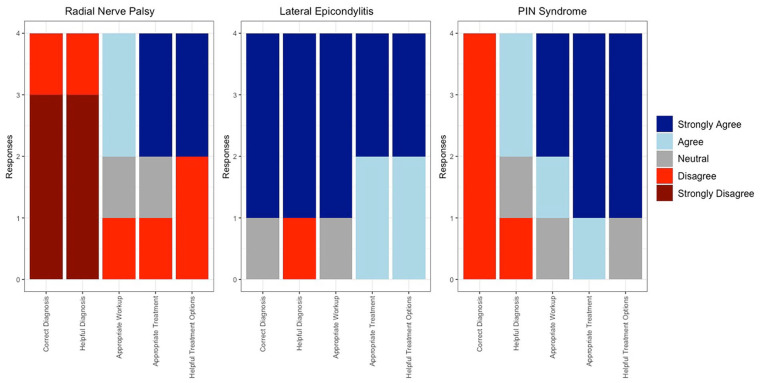
Stacked bar graph depicting individual surgeon responses to each vignette’s 4, 5, and 6 Likert survey.

**Figure 3.**
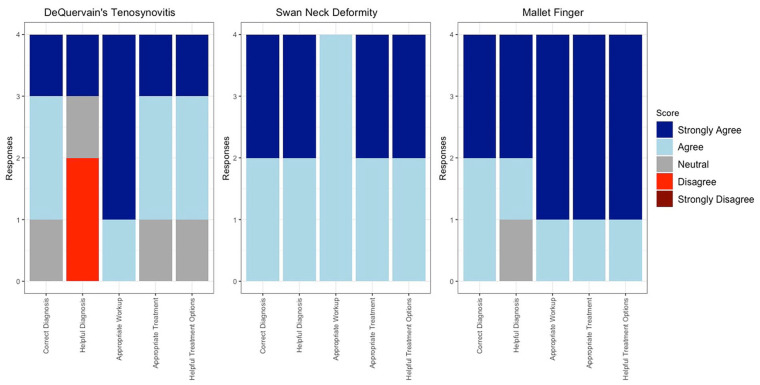
Stacked bar graph depicting individual surgeon responses to each vignette’s 7, 8, and 9 Likert survey.

Once ChatGPT responded to all the vignettes, 4 independently practicing fellowship-trained orthopedic hand surgeons scored ChatGPT’s response on 3 criteria: correctness (was the diagnosis, workup, and treatment correct), appropriateness (was the workup and treatment appropriate for the patient in the vignette), and helpfulness (was the diagnosis and treatment helpful for general orthopaedist, generalist, or hand surgeon who may not have experience with the pathology). They used a Likert scale ranging from 5 (*strongly agree*) to 1 (*strongly disagree*), and scores were averaged between all fellowship-trained orthopedic hand surgeons across all 9 vignettes. After independently scoring the responses, the surgeons came together and discussed each response to generate a consensus statement on ChatGPT’s diagnosis, workup, and treatment. Any dissenting opinions from the surgeons are supported via citations to the relevant literature. The vignette for trigger finger and the associated response can be seen in Table 1. All vignettes were presented and scored in a similar fashion. All other vignettes and surgeon consensus can be found in supplemental tables (Supplemental Table 1, Supplemental Table 2, Supplemental Table 3, Supplemental Table 4, Supplemental Table 5, Supplemental Table 6) and individual responses for each vignette in Figures 1 to 3.

**Table 1.** Trigger Finger.

A 64-year-old female presents with 4 months of pain and stiffness of her right ring finger. Her past medical history includes hypertension and diabetes. She states that the symptoms have come on gradually, she denies previous injury to the finger. She has noticed lately that her finger “clicks” and “occasionally gets stuck” when she flexes and extends the finger. On physical examination, she has tenderness over the A1 pulley of her ring finger, and there is a palpable nodule on the flexor tendon. When ranging her ring finger through flexion and extension, it reproduces the pain. Radiographs of the right hand are normal.		
Surgeon-written question	ChatGPT response	Surgeon consensus
What are the top diagnoses and why?	Based on the patient’s presentation, the top two diagnoses to consider are: 1. Trigger finger (stenosing tenosynovitis): The patient’s symptoms of pain, stiffness, clicking, and occasional locking of the finger, along with tenderness over the A1 pulley and a palpable nodule on the flexor tendon, are characteristic of trigger finger. This condition is caused by inflammation and narrowing of the tendon sheath, leading to difficulty in the smooth gliding of the tendon. It can be associated with conditions such as diabetes and repetitive strain on the finger. 2. Dupuytren’s contracture: This is another potential diagnosis, though less likely given the patient’s presentation. Dupuytren’s contracture is characterized by the thickening and shortening of the palmar fascia, leading to the development of nodules and cords that can limit finger movement. The symptoms usually develop gradually, but the absence of a history of a previous injury and normal radiographs make it a less likely diagnosis in this case. Trigger finger is the most likely diagnosis due to the patient’s symptoms, physical examination findings, and associated medical conditions. However, it is essential to rule out other potential diagnoses and consult with a healthcare professional for a definitive diagnosis and appropriate management.	ChatGPT’s answers for a differential diagnosis in a clinical vignette for a trigger finger are reasonable. However, it lacked more likely pathologies than Dupuytren’s, including sagittal band insufficiency, lateral band subluxation, and arthritis. In addition, the reasoning from ChatGPT including that “the absence of a history of a previous injury and normal radiographs make it a less likely diagnosis in this case” uses incorrect logic. It is correct that in patients with a strong family history of Dupuytren’s disease, a penetrating injury has been described as a nidus for the progression of the contracture.^12^ However, neither trauma nor radiography is associated with Dupuytren’s disease more than the other pathologies.
How would you workup trigger finger?	Working up trigger finger primarily involves a detailed history and physical examination, as imaging is not typically required for diagnosis. Here’s a step-by-step approach: 1. History: a. Onset and duration of symptoms: Determine when the patient first noticed the pain, stiffness, or locking sensation in the affected finger(s) and whether it has been progressively worsening. b. Associated factors: Ask about any repetitive activities or occupational factors that could contribute to the development of trigger finger. c. Medical history: Inquire about any relevant medical conditions, such as diabetes, rheumatoid arthritis, or hypothyroidism, which may predispose the patient to trigger finger. d. Prior treatments: If the patient has sought treatment for the symptoms before, find out what interventions were used and whether they were effective. 2. Physical examination: a. Inspection: Look for any visible swelling, redness, or deformity of the affected finger(s). b. Palpation: Gently palpate the flexor tendon at the base of the affected finger (near the A1 pulley) to assess for tenderness or a palpable nodule. c. Range of motion: Have the patient flex and extend the affected finger(s) to evaluate for pain, clicking, or locking sensations during movement. d. Compare to the contralateral hand: Examine the non-affected hand to compare the findings and help identify any patterns or differences. Although imaging studies are not typically needed for diagnosing trigger finger, in certain cases where the diagnosis is unclear, an ultrasound may be useful to visualize the thickened pully or tendon and confirm the diagnosis. After completing the history and physical examination, if the clinical presentation is consistent with trigger finger, a healthcare provider can discuss the appropriate management options with the patient.	The workup is overall appropriate, correctly identifying that it is a diagnosis made clinically. Under imaging, again, it is correct that imaging is not routinely needed for diagnosis. It could have included magnetic resonance imaging (MRI) in addition to ultrasound.
How would you treat trigger finger?	Treatment for trigger finger can be conservative or surgical, depending on the severity and duration of the symptoms. The goal is to reduce inflammation, pain, and improve finger mobility. Here are the common treatment options for trigger finger: 1. Conservative management: a. Rest: Avoid or minimize activities that involve repetitive gripping or finger movements to reduce inflammation and irritation of the tendon. b. Splinting: A splint may be used to immobilize the affected finger in an extended position, particularly at night, to help alleviate symptoms and promote healing. c. Nonsteroidal anti-inflammatory drugs (NSAIDs): Over-the-counter medications such as ibuprofen or naproxen can help reduce pain and inflammation. d. Ice: Apply ice to the affected area for 10-15 minutes several times a day to help minimize inflammation and pain. e. Gentle range-of-motion exercises: These exercises can help maintain finger mobility and prevent joint stiffness. 2. Corticosteroid injections: If conservative management fails to provide relief, a healthcare provider may consider a corticosteroid injection into the tendon sheath. This can help reduce inflammation and improve symptoms in many cases. However, the effectiveness of injections may be reduced in patients with diabetes or with recurrent symptoms. 3. Surgical intervention: If conservative measures and corticosteroid injections fail to provide relief or if the finger remains persistently locked, surgical intervention may be considered. The most common procedure is called percutaneous trigger finger release or open trigger finger release. These procedures involve releasing the A1 pulley, which allows the tendon to glide more freely and resolves the triggering. The choice of treatment depends on the severity of the symptoms, the patient’s medical history, and the response to previous interventions. A healthcare provider will recommend the most appropriate treatment plan based on the patient’s individual needs.	Overall, the treatment plan for trigger finger is reasonable. However, there was discussion about including corticosteroid injections under conservative options. In addition, modalities such as ice may help with pain but are not actual treatments. Early morning stretches can also be included.

## Results

**Figure 4.**
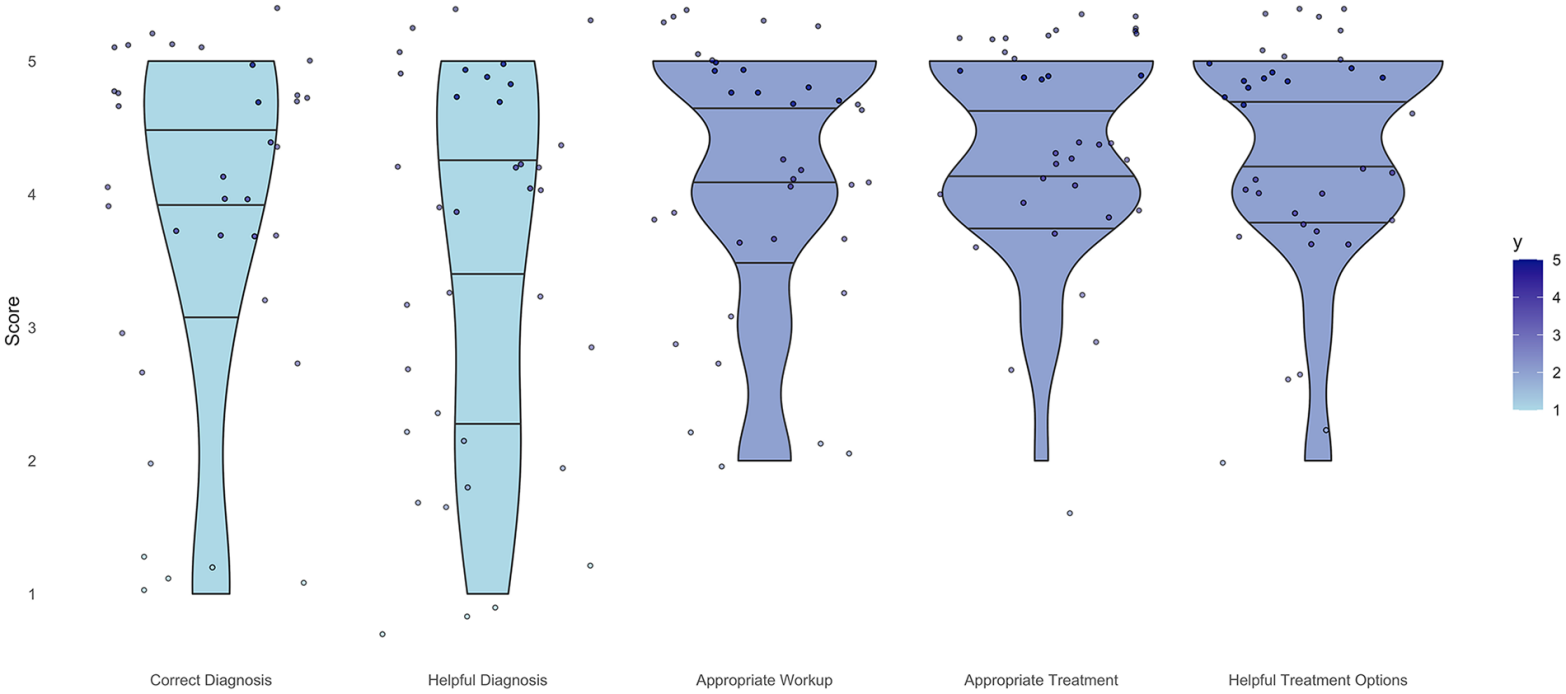
Violin plot of surgeon responses for each likert survey question demonstrating the spread of response over all questions.

ChatGPT accurately diagnosed 7 out of the 9 vignettes, resulting in a 78% accuracy rate. It misidentified sagittal band rupture (Table 2) and posterior interosseous nerve syndrome and failed to detect the incorrect nerve distribution in radial nerve palsy (Table 3). In terms of diagnostic performance, ChatGPT received a score of 3.8 ± 1.4. For guiding patient management, it scored 3.4 ± 1.4, while the appropriateness of workup for the actual diagnosis scored 4.1 ± 1.0. ChatGPT scored 4.3 ± 0.8 for an appropriate recommended treatment plan for the diagnosis, and 4.4 ± 0.8 for the helpfulness of treatment options in managing patients. Figure 4 shows the distribution of overall responses.

**Table 2.** Sagittal Band Rupture.

A 50-year-old female presents with a 4-month history of pain and inability to straighten her left long finger. Her past medical history includes rheumatoid arthritis. She denies any prior injury to the finger or hand. On physical examination of her left hand, there is mild swelling and tenderness over the long finger MCP joint. The long finger is held in a flexed position at the MCP joint at rest, and she is unable to actively extend the finger, but when the finger is passively extended, she is then able to hold it in extension. There is a snapping sensation when her finger is brought into extension. There is no tenderness over the A1 pulley and no palpable nodules. Her radiographs are unremarkable.		
Surgeon-written question	ChatGPT response	Surgeon responses
What are the most likely diagnoses?	Based on the patient’s presentation, the most likely diagnoses are: 1. Trigger finger (stenosing tenosynovitis): This condition occurs when inflammation narrows the space within the tendon sheath, causing the affected finger to catch or lock in a bent position. The snapping sensation when extending the finger and the ability to hold it in extension once passively extended are classic signs of trigger finger. Although it is more common in the A1 pulley, it can involve other areas. 2. Flexor tendon adhesion: Due to the patient’s history of rheumatoid arthritis, it is possible that inflammation has led to adhesion formation within the flexor tendon sheath, limiting the ability to actively extend the finger. 3. Joint synovitis related to rheumatoid arthritis: As the patient has a history of rheumatoid arthritis, it is possible that the pain and limited mobility are due to synovitis in the MCP joint. This could also contribute to the flexed position at rest and difficulty extending the finger. It is important to consult with a healthcare professional to determine the most accurate diagnosis and appropriate treatment options for the patient.	ChatGPT did not come to the appropriate diagnosis. Trigger finger is reasonable to include in the differential. Flexor tendon adhesion and joint synovitis are less likely.
Could sagittal band rupture be a part of the differential?	Yes, sagittal band rupture, also known as extensor tendon subluxation or dislocation, can be included in the differential diagnosis, although it is less likely given the patient’s presentation. Sagittal band rupture occurs when the sagittal band, which stabilizes the extensor tendon over the metacarpophalangeal (MCP) joint, is damaged or torn. This can result in the extensor tendon subluxating or dislocating from its normal position, leading to difficulty in extending the affected finger. The patient may have pain, swelling, and tenderness over the MCP joint, similar to this patient’s presentation. However, some key features of sagittal band rupture that are not present in this patient’s case include: 1. The snapping sensation is typically more pronounced during active extension, whereas in this case, the patient is unable to actively extend the finger. 2. Patients with sagittal band rupture usually report a history of trauma or injury to the hand or finger, which this patient denies. While it is less likely in this case, sagittal band rupture should not be completely ruled out without further examination and consultation with a healthcare professional.	The answer provided by ChatGPT failed to acknowledge atraumatic causes of sagittal band insufficiency, usually associated with underlying inflammatory causes. In addition, it did not understand the nuance between the clinical presentation of patients who cannot actively initiate MCP joint extension with complete dislocations of the extensor tendon.^13^
What is the workup for sagittal band rupture?	A workup for sagittal band rupture typically involves a combination of a thorough history, physical examination, and, if necessary, imaging studies. The goal is to identify the cause of the patient’s symptoms and rule out other potential diagnoses. 1. History: Obtain a detailed history of the patient’s symptoms, including the onset, duration, and any aggravating or alleviating factors. Ask about any history of trauma or repetitive stress to the affected hand, as well as any underlying medical conditions like rheumatoid arthritis. 2. Physical examination: Assess the range of motion, strength, and stability of the affected finger and its surrounding joints. Key findings in sagittal band rupture include the “buttonhole” deformity (also known as boutonniere deformity) and the subluxation of the extensor tendon to the ulnar or radial side of the affected metacarpophalangeal (MCP) joint. Some specific tests for sagittal band rupture during physical examination include: -Elson’s test: With the patient’s hand resting on a table and the MCP joint flexed to 90 degrees, the examiner applies resistance to the fingertip, attempting to further flex the proximal interphalangeal (PIP) joint. A positive test is indicated by the inability to extend the PIP joint against resistance. -Passive extension test: With the MCP joint in full flexion, the examiner attempts to passively extend the PIP joint. If the extensor mechanism is intact, passive extension should be possible. 3. Imaging studies: Imaging studies are not always necessary for diagnosing sagittal band rupture, but they may be helpful in certain cases or to rule out other diagnoses. X-rays can help identify any underlying joint abnormalities, fractures, or dislocations, while ultrasound or magnetic resonance imaging (MRI) may be useful for assessing soft tissue damage or inflammation. 4. Referral to a specialist: In cases where the diagnosis remains unclear or for management of more complex cases, a referral to a hand specialist, such as an orthopedic surgeon or a hand therapist, may be necessary. It is crucial to accurately diagnose a sagittal band rupture to determine the most appropriate treatment plan for the patient.	The response provided by ChatGPT was unexpected and also incorrect. It has confused the pathologies of sagittal band rupture and central slip injuries. The description of a boutonniere deformity and physical findings of Elson’s test describes a central slip injury.^14^ In addition, the explanation of an Elson’s test is incorrect. Generally, the examiner flexes the PIP 90° and then has the patient attempt active extension. Laxity of the distal interphalangeal (DIP) joint is then assessed with a more rigid DIP suggesting a central slip injury. In addition, we are unsure what the passive extension test is. It appears to be an inaccurate description or a fabricated exam. The logic described needs to be more consistent. For example, the description of the workup of a sagittal band rupture acknowledges a history of rheumatoid arthritis; however, the previous question of including sagittal band rupture in the differential failed to acknowledge nontraumatic causes.
How would you treat sagittal band rupture?	The treatment of sagittal band rupture depends on the severity and chronicity of the injury, as well as the patient’s overall health and functional demands. Treatment options can be divided into non-surgical and surgical approaches. 1. Non-surgical treatment: For acute, partial, or minimally displaced sagittal band rupture, conservative management may be sufficient. This typically involves: a. Immobilization: Use of a splint or orthosis to immobilize the affected finger in extension at the metacarpophalangeal (MCP) joint for 4-6 weeks. This helps to reduce strain on the injured sagittal band and allows it to heal. b. Anti-inflammatory medications: Over-the-counter or prescription nonsteroidal anti-inflammatory drugs (NSAIDs) can be used to manage pain and inflammation. c. Rest and activity modification: Avoiding activities that cause pain or stress to the affected finger, especially during the healing process. d. Physical therapy: once the immobilization. Is over, a hand therapist can guide the patient through a tailored rehabilitation program that focuses on improving range of motion, strength, and function of the affected finger. 2. Surgical treatment: If conservative management fails or if the sagittal band rupture is complete, displaced, or chronic, surgical intervention may be required. The goals of surgery are to repair the damage sagittal band, restore extensor tendon alignment, and improve finger function. Various surgical techniques can be used, such as direct repair, tendon transfers, or reconstruction of the sagittal band using graphs. The choice of technique depends on the specific nature of the injury and the surgeon’s expertise. Post-surgical care typically involves: a. Immobilization: A splint or orthosis is used to protect the surgical site and maintain proper alignment of the extensor tendon during the initial healing phase. b. Physical therapy: Once cleared by the surgeon, a hint therapist can help the patient with the rehabilitation program, focusing on regaining range of motion, strength, and function of the affected finger. c. Follow-up appointments: Regular follow up appointments with the surgeon or essential to monitor the progress of healing and ensure a successful outcome. It is important to consult with the healthcare professional to determine the most appropriate treatment plan for a patient with sagittal band rupture.	The management is reasonable with appropriate nonoperative management but vague operative treatment options.

*Note.* MCP = metacarpophalangeal; PIP = proximal interphalangeal; NSAIDs = nonsteroidal anti-inflammatory drugs; DIP = distal interphalangeal.

**Table 3.** Radial Nerve Palsy.

A 65-year-old female presents for follow-up after sustaining a right distal third humerus fracture. She initially presented to the emergency department 2 days ago where she underwent closed reduction and immobilization of the fracture. Since then, she has noticed gradual onset of weakness, numbness, and tingling of her forearm, wrist, and hand. On physical examination, there is weakness with wrist and thumb extension, as well as with finger extension at the metacarpophalangeal joints. She is able to flex and extend the fingers at the interphalangeal joints. She endorses numbness and tingling of the dorsal forearm, dorsum of the hand, as well as over the small, ring, and long fingers. She has palpable radial and ulnar pulses with brisk capillary refill to all digits. Radiographs reveal a spiral fracture of the distal third of the right humerus.		
Surgeon-written question	ChatGPT response	Surgeon responses
What are the most likely diagnoses?	The patient’s symptoms of weakness, numbness, and tingling in the forearm, wrist, and hand, as well as the physical examination findings, suggest a possible nerve injury. Based on the location of the fracture (distal third of the humerus) and the specific motor and sensory deficits, the most likely diagnosis is a radial nerve injury. The radial nerve is responsible for wrist and thumb extension, as well as finger extension at the metacarpophalangeal joints, which are the areas of weakness noted in the patient. Furthermore, the radial nerve provides sensory innervation to the dorsal forearm, dorsum of the hand, and the small, ring, and long fingers, which correspond to the patient’s reported numbness and tingling. In this case, the radial nerve may have been injured during the initial fracture or as a result of the closed reduction and immobilization. It is important to monitor the patient’s symptoms and consider further investigation, such as nerve conduction studies or electromyography, to assess the extent of the injury. Consultation with an orthopedic or hand surgeon may be warranted to discuss potential treatment options, including surgical exploration and repair if necessary.	The answer provided by ChatGPT alludes to a radial nerve injury associated with the distal humerus fracture. However, it did not provide a differential of other pathologies. Of note, ChatGPT did not acknowledge the inconsistent sensory distribution in the clinical vignette.
How would you workup radial nerve palsy?	A thorough workup of radial nerve palsy involves taking a detailed history, performing a physical examination, and utilizing appropriate diagnostic tests. Here is a suggested approach: 1. History: Obtain a detailed history of the patient’s symptoms, onset, and any preceding events, such as trauma or injury, which may have caused the radial nerve palsy. Ask about any associated pain, previous nerve problems, or systemic illnesses (like diabetes) that could predispose them to nerve injuries. 2. Physical examination: perform a comprehensive neurological and musculoskeletal examination, focusing on the affected upper extremity. Assess muscle strength, sensation, reflexes, and coordination. Evaluate the patient’s ability to perform specific movements, such as wrist and thumb extension, finger extension at the metacarpophalangeal joints, and finger flexion and extension at the interphalangeal joints. Check for any areas of sensory loss or altered sensation in the radial nerve distribution. 3. Diagnostic tests: a. Imaging: Obtain radiographs of the affected limb to evaluate for any fractures, dislocations, or other bony abnormalities that could be causing nerve compression. In some cases, additional imaging such as MRI or CT scans may be helpful to further assess soft tissue structures or bony details. b. Nerve conduction studies (NCS) and electromyography (EMG): These tests assess the function and integrity of the peripheral nerves and muscles. NCS can help identify the location and severity of the nerve injury, while EMG can provide information about muscle denervation and the potential for recovery. c. Blood tests: Depending on the patient’s history and risk factors, laboratory tests may be ordered to rule out underlying medical conditions that could contribute to nerve injury, such as diabetes, vitamin deficiencies, or autoimmune disorders. 4. Consultation with specialists: In cases of radial nerve palsy it may be necessary to involve specialists such as orthopedic surgeons, hand surgeons, or neurologists to help guide management and treatment decisions. 5. Monitor progress: Regular follow-ups are important to monitor the patient’s progress, assess recovery, and modified treatment plans as needed. This may include physical therapy to maintain range of motion and prevent muscle atrophy during the recovery process.	The workup overall is reasonable. The CT or MRI is generally not warranted, and the diagnosis is clinical. The timing of when an EMG/NCS is performed should have been mentioned.^15^
How would you treat radial nerve palsy?	The treatment of radial nerve palsy depends on the cause, severity, and duration of the nerve injury. A comprehensive management plan may include conservative measures, physical therapy, and surgical intervention, depending on the patient specific situation. Here are some general treatment options: 1. Conservative management: a. Observation: in cases of mild injury or compression, the nerve may recover spontaneously over time period it is important to monitor the patient’s symptoms and progress closely. b. Pain management: prescribe appropriate analgesics, such as acetaminophen, NSAIDs, or neuropathic pain medications (like gabapentin or pregabalin), to manage pain associated with nerve injury. c. Immobilization: in cases of acute injury, a splint or brace may be used to provide support and maintain proper alignment of the wrist and hand. This can help minimize further nerve injury and promote healing. 2. Physical therapy: a. Range of motion exercises: To prevent stiffness and maintain joint mobility, patients should be encouraged to perform gentle range of motion exercises. b. Strengthening exercises: As the nerve begins to recover, targeted strengthening exercises can be introduced to help regain muscle function. c. Occupational therapy: Patients may benefit from occupational therapy to help them adapt to any functional limitations and learn techniques to perform daily tasks. 3. Surgical intervention: a. Nerve decompression: If the radial nerve palsy is caused by compression from a tight cast or external pressure, surgical decompression may be necessary to relieve pressure on the nerve. b. Nerve repair or grafting: In cases of severe nerve injury or laceration, surgical repair or nerve grafting may be required to restore function. The prognosis for recovery depends on the extent of the injury, the quality of the repair, and the duration of the nerve injury. c. Tendon transfers: In cases of irreversible nerve injury or long standing palsy, tendon transfer surgery may be considered to restore function to the affected muscles. This involves transferring a functional tendon from another muscle to the paralyzed muscle to the restorative movement. 4. Follow up and monitoring: Regular follow-ups are crucial to assess recovery and adjust treatment plans accordingly. The duration of recovery can vary widely, depending on the severity of the injury and the patient’s overall health. It is important to tailor the treatment plan to the individual patient’s needs, taking into account the cause, severity, and duration of the radial nerve palsy, as well as the patient’s overall health and functional goals. Evolvement of specialists such as orthopedic surgeons, hand surgeons, or neurologists may be necessary for optimal management.	The listed treatment options for radial nerve palsy are generally appropriate management for isolated cases of radial nerve palsy. It did not address the distal humerus fracture. However, it was not prompted to do so. In the nuanced case of a radial nerve palsy with a nonoperative distal humerus fracture, the management is generally conservative, given the likelihood of nerve function recovery without surgery.^16,17^

*Note.* MRI = magnetic resonance imaging; CT = computed tomography; NCS = nerve conduction studies; EMG = electromyography; NSAIDs = nonsteroidal anti-inflammatory drugs.

## Discussion

Seth et al^5^ assessed ChatGPT’s ability to provide general information about carpal tunnel syndrome to patients and suggested that LLMs could potentially serve as a surrogate medical practitioner or supportive tools for clinicians. This study assessed the ability of ChatGPT to assist a clinician in generating a differential diagnosis, selecting appropriate workup, and treatment when presented with 9 vignettes representing a diverse set of hand pathology. Overall, ChatGPT correctly identified the correct diagnosis 7 out of 9 times (78% correct), was generally successful in explaining potential workups, and was able to explain both operative and nonoperative treatment options for a variety of conditions. Importantly, the ChatGPT failed to identify an atypical presentation of radial nerve palsy, where the distribution described was inconsistent with the radial nerve. This is cause for concern, as it suggests the ChatGPT may be unable to detect the nuances necessary to provide effective and safe care. While ChatGPT excelled in many areas, demonstrating tremendous promise for integration into the clinic, its utility to a fellowship-trained surgeon may be limited in the clinic.

Our findings are similar to published findings in other disciplines. Seth et al^5^ evaluated ChatGPT’s ability to disseminate clinically relevant information to patients in the context of carpal tunnel syndrome. They concluded that while ChatGPT has the capacity to convey medical information to patients, it is restrained by its indiscretion in supplying nonexistent and inaccurate references and information. Grünebaum et al^18^ assessed ChatGPT’s responses to various queries related to obstetrics and gynecology. They noted that, while answers were frequently well-constructed and reasonable, ChatGPT often demonstrated a lack of insight and inability to grasp the nuance of both technical questions and more complex, abstract aspects of human language. Barat et al,^19^ who assessed ChatGPT’s ability to provide guidance in interventional radiology, found that ChatGPT provided correct answers only 40%. While ChatGPT was able to generate coherent, plausible responses, many answers lacked the nuance or specificity required to be useful in the clinical setting.

Other studies have assessed ChatGPT’s ability to educate patients and have produced promising, but similarly mixed results. Juhi et al^20^ assessed ChatGPT’s ability to provide patients advice regarding medications and drug interactions. While ChatGPT was able to identify 39 out of 40 drug interactions, the authors noted that many explanations were inconclusive or unclear. Furthermore, ChatGPT was able to answer basic-level questions about diabetes and diabetes care^21^; however, another study noted several inaccuracies and limitations, such as the stochasticity of AI-generated responses and the endpoint of training data.^22^ Interestingly, when comparing ChatGPT’s ability to answer frequently asked questions in arthroplasty, Dubin et al^23^ found that ChatGPT more frequently sourced answers from government or academic publications relative to Google, which provided more information from commercial sources. Still, they did not rigorously assess the veracity of the answers, and simply concluded that patients should corroborate this information with their physician.

While our assessment was generally favorable, there are several important limitations of ChatGPT that may hinder its incorporation into the clinic. First, ChatGPT’s knowledge is only as comprehensive and accurate as the data it was trained on. Importantly, the current iteration of ChatGPT is only trained on data up to September 2021; therefore, it may lack information on more recent literature and advances. Furthermore, it is a generally trained LLM, meaning it lacks medical and orthopedic specific training data, potentially hindering its performance. However, this does make the possibility of an orthopedic-trained ChatGPT more enticing, as the generally trained ChatGPT performed impressively without specific training.

Second, “hallucinations,” a seemingly confident, coherent response generated by ChatGPT that is not backed by its training data, are common. More plainly, ChatGPT may generate a response to an objective question that is fabricated but presents it as factual. Not only are there concerns regarding the origin and implications of these hallucinations, but the cogent way this false information is presented compounds the issue. Both clinicians and patients need to be aware of this potential and ensure the correctness of ChatGPT’s recommendations with a credible source.

Finally, recommendations or evidence provided by ChatGPT are difficult, or impossible, to validate. While it presents information in a clear, concise manner, when asked to provide references, it will fabricate citations.^24^ These citations, to an unsuspecting reader, will appear legitimate, but titles and identifiers are frequently falsified or incorrectly identify a source for the material. Furthermore, these false references can bolster the perceived validity of false, biased, or otherwise incorrect information, creating concerns for patient safety.

Our study has a number of important limitations. First, we primarily assessed hand pathology. While ChatGPT is ostensibly trained on general knowledge, the specific scope and breadth of its knowledge is unclear. It is not possible to generalize our findings to other fields; therefore, further testing of the AI chatbot is still warranted in other disciplines and subspecialities. Second, our assessment only included relatively short, text-based vignettes, many with textbook presentations. In clinical practice, imaging, the physical exam, and history taking skills are all important components of patient care. Further research is still required to assess ChatGPT’s ability to incorporate and synthesize multimodal streams of information that may not be as clear. Third, our assessment of ChatGPT’s performance is limited, both due to the subjectivity of the evaluation process and also due to lack of comparison. Future work may draw stronger conclusions by comparing ChatGPT’s performance to that of hand surgeons. Finally, our study was limited to only 9 vignettes and did not have a comparison or control group. Future studies can build upon this work by testing a greater sample of vignettes and incorporating a comparison or control group.

## Conclusion

Given our findings, ChatGPT is likely not a useful resource for surgeons in the clinic at this time. Although ChatGPT successfully diagnosed multiple conditions in hand surgery based on clinical vignettes, it made multiple errors. While, in the future, there may be a role for natural language models in clinics, further work and technological advancements are still necessary to safely incorporate ChatGPT or similar technologies into patient care. This study cannot endorse the use of ChatGPT in the clinical setting at this time.

## Supplemental Material

sj-docx-1-han-10.1177_15589447241257643 – Supplemental material for Evaluation of a Large Language Model’s Ability to Assist in an Orthopedic Hand Clinic Supplemental material, sj-docx-1-han-10.1177_15589447241257643 for Evaluation of a Large Language Model’s Ability to Assist in an Orthopedic Hand Clinic by Travis Kotzur, Aaron Singh, John Parker, Blaire Peterson, Brian Sager, Ryan Rose, Fred Corley and Christina Brady in HAND

sj-docx-2-han-10.1177_15589447241257643 – Supplemental material for Evaluation of a Large Language Model’s Ability to Assist in an Orthopedic Hand Clinic Supplemental material, sj-docx-2-han-10.1177_15589447241257643 for Evaluation of a Large Language Model’s Ability to Assist in an Orthopedic Hand Clinic by Travis Kotzur, Aaron Singh, John Parker, Blaire Peterson, Brian Sager, Ryan Rose, Fred Corley and Christina Brady in HAND

sj-docx-3-han-10.1177_15589447241257643 – Supplemental material for Evaluation of a Large Language Model’s Ability to Assist in an Orthopedic Hand Clinic Supplemental material, sj-docx-3-han-10.1177_15589447241257643 for Evaluation of a Large Language Model’s Ability to Assist in an Orthopedic Hand Clinic by Travis Kotzur, Aaron Singh, John Parker, Blaire Peterson, Brian Sager, Ryan Rose, Fred Corley and Christina Brady in HAND

sj-docx-4-han-10.1177_15589447241257643 – Supplemental material for Evaluation of a Large Language Model’s Ability to Assist in an Orthopedic Hand Clinic Supplemental material, sj-docx-4-han-10.1177_15589447241257643 for Evaluation of a Large Language Model’s Ability to Assist in an Orthopedic Hand Clinic by Travis Kotzur, Aaron Singh, John Parker, Blaire Peterson, Brian Sager, Ryan Rose, Fred Corley and Christina Brady in HAND

sj-docx-5-han-10.1177_15589447241257643 – Supplemental material for Evaluation of a Large Language Model’s Ability to Assist in an Orthopedic Hand Clinic Supplemental material, sj-docx-5-han-10.1177_15589447241257643 for Evaluation of a Large Language Model’s Ability to Assist in an Orthopedic Hand Clinic by Travis Kotzur, Aaron Singh, John Parker, Blaire Peterson, Brian Sager, Ryan Rose, Fred Corley and Christina Brady in HAND

sj-docx-6-han-10.1177_15589447241257643 – Supplemental material for Evaluation of a Large Language Model’s Ability to Assist in an Orthopedic Hand Clinic Supplemental material, sj-docx-6-han-10.1177_15589447241257643 for Evaluation of a Large Language Model’s Ability to Assist in an Orthopedic Hand Clinic by Travis Kotzur, Aaron Singh, John Parker, Blaire Peterson, Brian Sager, Ryan Rose, Fred Corley and Christina Brady in HAND
