# Supplementary material for: Evaluation of a Large Language Model’s Ability to Assist in an Orthopedic Hand Clinic
Source: Hand (N Y). 2024 Jun 22;20(6):900–9. doi: 10.1177/15589447241257643 (PMC11571334; doi:10.1177/15589447241257643)
Supplement: sj-docx-1-han-10.1177_15589447241257643 – Supplemental material for Evaluation of a Large Language Model’s Ability to Assist in an Orthopedic Hand Clinic [file sj-docx-1-han-10.1177_15589447241257643.docx]

| **Supplemental Table 1 \| Dupuytren’s** | | |
| --- | --- | --- |
| A 60-year-old male presents to your office with 1 year of pain and stiffness of his right ring and small fingers. His past medical history includes diabetes mellitus. He states that the symptoms have come on gradually, he denies previous injury to the fingers or hand. He states that his ring and small fingers are becoming “stuck” and he “cannot straighten them”. On physical examination he has decreased range of motion of the ring and small fingers and they are contracted. There are painful nodules in the palm. When asked to place his palm flat on the table the ring and small fingers remain in the flexed position. Radiographs of the right hand are normal. | | |
| **Surgeon-written question** | **ChatGPT response** | **Surgeon responses** |
| What are the most likely diagnoses? | Based on the patient’s history, symptoms, and physical examination, the most likely diagnosis is Dupuytren’s contracture. Dupuytren’s contracture is a progressive condition in which fibrous tissue (palmar fascia) in the palm of the hand thickens and tightens, causing one or more fingers to bend into the palm. The exact cause of Dupuytren’s contracture is unknown, but risk factors include age, male gender, and a history of diabetes mellitus.  The patient’s symptoms of pain and stiffness in the right ring and small fingers, the presence of painful nodules in the palm, and the inability to place the palm flat on the table with the affected fingers remaining in a flexed position are all consistent with Dupuytren’s contracture. Additionally, the normal radiographs of the right hand support this diagnosis, as Dupuytren’s contracture typically does not cause bony abnormalities.  Other potential diagnoses to consider in this case might include trigger finger, which is characterized by a finger getting stuck in a bent position and then suddenly straightening with a snap, and tendonitis or tenosynovitis, which could cause pain and limited finger movement. However, the presence of nodules, contracture of the fingers, and the patient’s risk factors make Dupuytren’s contracture the most likely diagnosis. | ChatGPT got the diagnosis correct with Dupuytren’s. However, it did not include other contracture causes, such as PIP contracture secondary to trauma. In addition, chat GPT did not comment on the nodules described as painful, which the surgeons noted since once contractures develop in Dupuytrens patients, the nodules are usually no longer painful^1^. |
| How would you workup Dupuytren’s contracture? | Dupuytren’s contracture is primarily a clinical diagnosis based on the patient’s history, symptoms, and physical examination. In most cases, no additional tests are needed. However, a proper workup for Dupuytren’s contracture should include the following steps:  1. Detailed medical history: Obtain a thorough medical history from the patient, focusing on the onset, duration, and progression of symptoms. Ask about any previous hand injuries or surgeries, as well as family history of Dupuytren’s contracture, since there is a hereditary component to the disease.  2. Physical examination: Examine the patient’s hand, checking for the presence of nodules or cords in the palm and any contractures of the fingers. Assess the range of motion of the affected fingers and test the patient’s grip strength. Perform the “tabletop test” by asking the patient to place their palm flat on a table. Inability to do so due to the flexed position of the affected fingers is indicative of Dupuytren’s contracture.  3. Imaging studies: Although imaging is not typically required for the diagnosis of Dupuytren’s contracture, plain radiographs (X-rays) of the hand can help rule out other potential causes of the patient’s symptoms, such as arthritis or fractures.  4: Referral to a specialist: If the diagnosis is unclear or if the patient requires surgical intervention, referral to a hand surgeon or orthopedic specialist with experience in treating Dupuytren's contracture may be necessary.  Ultimately, the workup for Dupuytren’s contracture focuses on a comprehensive medical history and physical examination. Imaging studies may be used to rule out other diagnoses, and referral to a specialist can be considered for further evaluation and management. | Workup for Dupuytren’s is appropriate with emphasis of a diagnosis made clinically and positive family history. |
| How would you treat Dupuytren’s contracture? | The treatment for Dupuytren’s contracture depends on the severity of the condition and the impact on the patient’s daily activities. Treatment options include:  1. Observation: In mild cases, where the contracture does not significantly affect hand function or cause pain, no treatment may be needed, and the condition can be monitored over time.  2. Physical therapy and splinting: A hand therapist can provide a customized stretching and strengthening program to help maintain hand function and prevent further contracture. In some cases, wearing a splint at night may help keep the affected fingers straight and prevent progression of the contracture.  3. Non-surgical treatment options: a. Enzyme injections (collagenase Clostridium histolyticum): This enzyme can be injected into the affected tissue to break down the collagen fibers causing the contracture, which allows the finger to be straightened during a subsequent in-office procedure called a finger extension. b. Needle aponeurotomy (percutaneous needle fasciotomy): A needle is used to puncture and weaken the contracted tissue, allowing the finger to be straightened. This procedure is less invasive than surgery, but the risk of contracture recurrence may be higher.  4. Surgical treatment options: a. Limited fasciectomy: This is the most common surgical procedure for Dupuytren’s contracture. The surgeon removes the affected tissue (palmar fascia) to allow the fingers to straighten. This procedure has a lower risk of recurrence but requires a longer recovery period. b. Dermofasciectomy: This procedure involves removing both the affected palmar fascia and the overlying skin. A skin graft is then used to cover the area. This method is typically reserved for severe or recurrent cases, as it has a lower recurrence rate but a more extended recovery period and higher risk of complications.  5. Postoperative care: After surgical treatment, hand therapy and splinting may be necessary to optimize recovery and maintain hand function.  The choice of treatment depends on the severity of the contracture, patient preferences, and the potential risks and benefits of each option. A consultation with a hand surgeon or an orthopedic specialist experienced in treating Dupuytren’s contracture can help guide the decision-making process. | Overall, there is agreement that the treatment options included are accurate. However, it does not specify the subcategories of when each treatment is most appropriate. It also does not list the augmentation option with a digit widget with more complex PIP contractures. There was a discussion of the efficacy of stretching with Dupuytrens^1^.  There was discussion about whether collagenase and needle aponeurotomy are “non-surgical” or should have been categorized as in-office procedures. Small details in the description with collagenase describe the manipulation of the finger as “finger extension,” which is an odd description. |

1. What Are the Best Diagnostic Criteria for Lateral Epicondylitis? Clinical Gate. Published March 16, 2015. Accessed May 21, 2023. https://clinicalgate.com/what-are-the-best-diagnostic-criteria-for-lateral-epicondylitis/
