# Supplementary material for: Evaluation of a Large Language Model’s Ability to Assist in an Orthopedic Hand Clinic
Source: Hand (N Y). 2024 Jun 22;20(6):900–9. doi: 10.1177/15589447241257643 (PMC11571334; doi:10.1177/15589447241257643)
Supplement: sj-docx-2-han-10.1177_15589447241257643 – Supplemental material for Evaluation of a Large Language Model’s Ability to Assist in an Orthopedic Hand Clinic [file sj-docx-2-han-10.1177_15589447241257643.docx]

| **Supplemental Table 2 \| Lateral Epicondylitis** | | |
| --- | --- | --- |
| A 45-year-old right hand dominant female presents with 2 months of right elbow pain. Her past medical history is unremarkable. She denies previous injury to the right elbow. She is active and enjoys playing tennis. On physical examination there is no swelling or erythema of the elbow. There is tenderness to palpation over the lateral epicondyle. With the elbow in full extension, the pain over the lateral elbow is reproducible with resisted wrist extension and maximal flexion of the wrist. She denies numbness or tingling in the forearm or hand. Radiographs of the right elbow are unremarkable. | | |
| **Surgeon-written question** | **ChatGPT response** | **Surgeon responses** |
| What are the most likely diagnoses? | Based on the patient's history, physical examination, and negative radiographs, the most likely diagnosis is lateral epicondylitis, also known as tennis elbow. This condition is characterized by pain and tenderness over the lateral epicondyle of the humerus and is commonly associated with repetitive activities involving risk extension, such as playing tennis. Lateral epicondylitis results from degeneration and microtears on the extensor tendon origin at the lateral epicondyle, leading to inflammation and pain. The pain is typically exacerbated by resisted wrist extension and gripping activities. The absence of swelling, erythema, and neurological symptoms (numbness or tingling) further supports this diagnosis. | Chat GPT got the diagnosis correct as lateral epicondylitis. However, it did not include other pathologies such as radial tunnel syndrome, radiculopathy, or intra-articular pathologies such as plica or osteochondral defects. Of note, it had a better differential in the answer for the workup of lateral epicondylitis. |
| How would you workup lateral epicondylitis? | The workup for lateral epicondylitis typically involves a thorough history, physical examination, and, in some cases, additional imaging studies. Here are the steps to follow:  1. History: Obtain a detailed history of the patient symptoms, including the onset, duration, and intensity of pain, as well as aggravating and relieving factors. Asking about activities that may contribute to the condition, such as sports or occupation related repetitive motions.  2. Physical examination: Perform a comprehensive examination of the affected elbow, including inspection, palpation, range of motion, and specific provocative tests. In lateral epicondylitis, tenderness over the lateral epicondyle, pain with resisted wrist extension, and pain with gripping activities are common findings.  3. Provocative tests: The Cozen’s test (resisted wrist extension with the elbow in full extension) and the Mill’s test (passive wrist flexion with the elbow in full extension) can help confirm the diagnosis.  4. Imaging studies: While plain radiographs are often normal in lateral epicondylitis, they can be useful to rule other causes of elbow pain, such as fractures or arthritis. In some cases, ultrasound or magnetic resonance imaging (MRI) may be indicated to further evaluate the tendons and soft tissues around the elbow, especially if conservative treatment fails or if other conditions are suspected.  5. Differential diagnosis: It's important to consider and rule out other possible causes of lateral elbow pain, such as radial tunnel syndrome, posterolateral rotatory instability, or osteochondritis dissecans.  After a thorough workup, if lateral epicondylitis is confirmed, the focus shifts to management and treatment. | ChatGPT included an appropriate workup for lateral epicondylitis.  The descriptions of the Cozen and Mills test were inaccurate. ChatGPT wrote that the Cozen's test is "resisted wrist extension with the elbow in full extension," however, the described examination is with the elbow flexed to 90 degrees with the forearm in full pronation with resisted wrist extension. For Mill's test, ChatGPT described it as "passive wrist flexion with the elbow in full extension," which is mostly correct, except the hand should be closed, the wrist in dorsiflexion with the elbow extended, and resisted wrist flexion. A positive test is reproducing pain at the lateral epicondyle^1-3^. |
| How would you treat lateral epicondylitis? | The treatment of lateral epicondylitis (tennis elbow) typically involves conservative measures initially, with surgical intervention reserved for cases that do not respond to non-operative treatment. The goals of treatment are to reduce pain, promote healing, and restore function. Here are the steps involved in treating lateral epicondylitis:  1. Activity modification: Advise the patient to avoid or modify activities that exacerbate symptoms, such as repetitive wrist extension or gripping.  2. Rest: Encourage the patient to rest the affected arm, allowing time for the healing process.  3. Ice: Applying ice to the affected area can help reduce pain and inflammation. Recommend icing the elbow for 15-20 minutes, 3-4 times a day.  4. Non-steroidal anti-inflammatory drugs (NSAIDs): Over the counter NSAIDs, such as ibuprofen or naproxen, can help alleviate pain and reduce inflammation.  5. Physical therapy: A physical therapist can help the patient develop a rehabilitation program to strengthen the forearm and wrist muscles, improve flexibility, and promote proper biomechanics. This may include stretching exercises, eccentric strengthening exercises, and manual therapy techniques.  6. Counterforce bracing: Using a counterforce brace or forearm strap can help redistribute forces and alleviate pain by reducing stress on the tendon attachment.  7. Corticosteroid injections: In some cases, a corticosteroid injection may be considered for short term pain relief. However, multiple injections should be avoided as they may increase the risk of tendon rupture.  8. Extracorporeal shock wave therapy (ESWT): This treatment modality may be considered in some cases, though the evidence for its effectiveness and lateral epicondylitis is not yet conclusive.  9. Platelet rich plasma (PRP) injections: PPRP injections have shown some promise in treating lateral epicondylitis by promoting tendon healing, though further research is needed to establish their effectiveness.  If conservative treatments fail to provide relief after an appropriate period (usually 6 to 12 months), surgical intervention may be considered. Surgical options for lateral epicondylitis include open or orthoscopic procedures to remove the damaged tendon tissue and stimulate healing. The choice of surgery depends on the surgeon's preference, patient factors, and the extent of the pathology. Recovery and rehabilitation following surgery may take several weeks to months. | There is a consensus on appropriate treatment options by Chat GPT.  Multiple corticosteroid injections should be avoided, although the reasoning may not be due to tendon rupture^4,5^. It seemed aware of the ongoing debate with PRP, which has demonstrated promising results with specifically lateral epicondylitis^5,6^.  It failed to include wrist bracing. |

1. Sun JQ, Stillson QA, Strelzow JA, Shi LL. Trends in Corticosteroid Injections for Treatment of Lateral Epicondylitis: An Analysis of 80,169 Patients. J Am Acad Orthop Surg Glob Res Rev. 2021;5(9):e21.00186. doi:10.5435/JAAOSGlobal-D-21-00186
2. Behrens SB, Deren ME, Matson AP, Bruce B, Green A. A review of modern management of lateral epicondylitis. Phys Sportsmed. 2012;40(2):34-40. doi:10.3810/psm.2012.05.1963
3. Ahmad Z, Brooks R, Kang SN, et al. The effect of platelet-rich plasma on clinical outcomes in lateral epicondylitis. Arthrosc J Arthrosc Relat Surg Off Publ Arthrosc Assoc N Am Int Arthrosc Assoc. 2013;29(11):1851-1862. doi:10.1016/j.arthro.2013.07.272
4. Dang AC, Rodner CM. Unusual compression neuropathies of the forearm, part I: radial nerve. J Hand Surg. 2009;34(10):1906-1914. doi:10.1016/j.jhsa.2009.10.016
5. Moore JS. De Quervain’s tenosynovitis. Stenosing tenosynovitis of the first dorsal compartment. J Occup Environ Med. 1997;39(10):990-1002. doi:10.1097/00043764-199710000-00011
6. Wu F, Rajpura A, Sandher D. Finkelstein’s Test Is Superior to Eichhoff’s Test in the Investigation of de Quervain’s Disease. J Hand Microsurg. 2018;10(2):116-118. doi:10.1055/s-0038-1626690
