# Supplementary material for: Evaluation of a Large Language Model’s Ability to Assist in an Orthopedic Hand Clinic
Source: Hand (N Y). 2024 Jun 22;20(6):900–9. doi: 10.1177/15589447241257643 (PMC11571334; doi:10.1177/15589447241257643)
Supplement: sj-docx-3-han-10.1177_15589447241257643 – Supplemental material for Evaluation of a Large Language Model’s Ability to Assist in an Orthopedic Hand Clinic [file sj-docx-3-han-10.1177_15589447241257643.docx]

| **Supplemental Table 3 \| Posterior Interosseous Nerve Syndrome** | | |
| --- | --- | --- |
| A 40-year-old right hand dominant male presents with insidious onset of right forearm and wrist pain over the past 2 years. His past medical history is significant for hypertension and diabetes mellitus. He is a manual laborer and his symptoms are exacerbated while lifting heavy objects at work. He describes the pain as deep and aching. On physical examination there is tenderness to palpation over the forearm just distal to the lateral epicondyle. Strength testing reveals weakness with wrist, finger metacarpophalangeal joint, and thumb extension. When attempting wrist extension, you notice that it extends with radial deviation. Sensation is intact in the median, radial, and ulnar nerve distributions. The pain just distal to the lateral epicondyle is reproducible with resisted forearm supination. Radiographs of the right elbow, forearm, and wrist are unremarkable. | | |
| **Surgeon-written question** | **ChatGPT response** | **Surgeon responses** |
| What are the most likely diagnoses? | Based on the patient's history, occupation, and physical examination, the most likely diagnosis are:  Lateral epicondylitis (tennis elbow): This condition is characterized by pain and tenderness at the lateral epicondyle, which is worsened by resisted forearm supination and wrist extension. The patient's occupation as a manual laborer and the insidious onset of pain also point towards this diagnosis.  Radial tunnel syndrome: This is a less common condition caused by compression of the radial nerve, usually at the level of the supinator muscle. It can present with similar symptoms as lateral epicondylitis, such as pain in the forearm and weakness in wrist and finger extension. Radial tunnel syndrome can coexist with lateral epicondylitis in some cases.  Considering the patient's hypertension and diabetes, it's important to rule out other causes like peripheral neuropathy or cervical radiculopathy. Further evaluations, such as nerve conduction studies and electromyography, may be needed to confirm the diagnosis and differentiate between these conditions. | Posterior interosseous nerve syndrome is an esoteric diagnosis. Chat GPT correctly included the diagnosis of lateral epicondylitis and radial tunnel syndrome. Although radial tunnel syndrome and PIN syndrome describe compression of the radial nerve, the clinical presentations are different. Patients with PIN syndrome lose motor function, whereas patients with radial tunnel syndrome typically present with mobile wad and lateral forearm pain without motor involvement^1^. |
| How would you workup posterior interosseous nerve syndrome? | Posterior interosseous nerve syndrome (PINS) is a condition in which the posterior interosseous nerve, a branch of the radial nerve, is compressed, typically within the radial tunnel. It may present with wrist and finger extensor weakness without pain, unlike radial tunnel syndrome, which typically presents with pain. To work up PINS, consider the following steps:  1. Detailed history and physical examination: Assess the onset, duration, and pattern of symptoms, as well as any aggravating factors. Evaluate for tenderness, weakness, and sensation in the affected arm.  2. Nerve conduction studies (NCS) and electromyography (EMG): These tests can help confirm PINS by identifying nerve compression or entrapment and differentiating it from other conditions such as radial tunnel syndrome, lateral epicondylitis, or cervical radiculopathy.  3. Imaging studies: While not routinely indicated, magnetic resonance imaging (MRI) or ultrasound can help be helpful in visualizing the course of the posterior interosseous nerve and any potential compression points, such as within the supinator muscle (the arcade of Frohse) or at the level of the radial tunnel.  4. Differential diagnosis: It is important to rule out other causes of wrist and finger extensor weakness, such as cervical radiculopathy, peripheral neuropathy, or other nerve entrapment syndromes.  5. Conservative management: In many cases, PINS can be managed conservatively with rest, activity modification, anti-inflammatory medications, and physical therapy.  6. Surgical intervention: If conservative management fails or there is progressive neurological deficit, surgical decompression of the posterior interosseous nerve may be considered.  It is crucial to work closely with the specialist, such as a hand surgeon or neurologist, to accurately diagnose and manage PINS. | The description of differentiating PIN syndrome and radial tunnel syndrome is partially correct, with the main difference between the presence of motor weakness with PIN syndrome and not radial tunnel syndrome.  Other workups could have been mentioned, including advanced cervical spine imaging. |
| How would you treat posterior interosseous nerve syndrome? | The treatment for posterior interosseous nerve syndrome (PINS) typically begins with conservative management. However, if conservative treatment fails or there is a progressive neurological deficit, surgical intervention may be considered. Here is a stepwise approach to treating PINS:  1. Conservative management: a. Rest: Encourage the patient to rest the affected arm and avoid activities that exacerbate the symptoms. b. Activity modification: Adjust work or daily activities to minimize repetitive forearm motions or excessive strain on the affected arm. c. Non-steroidal anti-inflammatory drugs (NSAIDs): Over-the-counter or prescription and NSAIDs can help reduce inflammation and pain. d. Physical therapy: A physical therapist can provide a customized exercise program aimed at stretching and strengthening the affected muscles, as well as guidance on proper ergonomics and activity modification. e. Bracing or splinting: In some cases, wearing a brace or splint that supports the wrist and forearm may help alleviate symptoms. f. Corticosteroid injections: Though not typically used for PINS, corticosteroid injections may be considered in cases where radial tunnel syndrome or lateral epicondylitis are suspected to coexist.  2. Surgical intervention: If conservative management fails or there is progressive neurological deficit, surgical decompression of the posterior nauseous nerve may be necessary. The procedure typically involves releasing any points of compression along the nerve, such as the arcade of Frohse or fibrous bands within the supinator muscle.  It is essential to work closely with a specialist, such as a hand surgeon and neurologist, to accurately diagnose and manage PINS. A multidisciplinary approach, including physical therapy and occupational therapy, may be beneficial for optimal recovery and return to normal function. | The treatment for PIN syndrome is overall accurate.  It correctly mentions that progressive neurological symptoms may warrant surgical intervention.  It fails to acknowledge the use of diagnostic steroid injections in cases of PIN syndrome.  The surgical decompression sites correctly mention compression sites, including the arcade of Fröhse and the supinator muscle fibrous bands. It did not include the fibrous tissue anterior to the radiocapitellar joint between the brachialis and brachioradialis, the recurrent radial vessels /“leash of Henry,” or the proximal edge of the extensor carpi radialis brevis (ECRB)^1^. |

1. Lane R, Nallamothu SV. Swan-Neck Deformity. In: StatPearls. StatPearls Publishing; 2023. Accessed May 21, 2023. http://www.ncbi.nlm.nih.gov/books/NBK525970/
