# Supplementary material for: Evaluation of a Large Language Model’s Ability to Assist in an Orthopedic Hand Clinic
Source: Hand (N Y). 2024 Jun 22;20(6):900–9. doi: 10.1177/15589447241257643 (PMC11571334; doi:10.1177/15589447241257643)
Supplement: sj-docx-4-han-10.1177_15589447241257643 – Supplemental material for Evaluation of a Large Language Model’s Ability to Assist in an Orthopedic Hand Clinic [file sj-docx-4-han-10.1177_15589447241257643.docx]

| **Supplemental Table 4 \| De Quervain’s Tenosynovitis** | | |
| --- | --- | --- |
| A 45-year-old right hand dominant female presents with 1 month of right wrist pain. Her past medical history is unremarkable. She reports that she is frequently using her phone for texting and sending emails for her job. On physical examination of the right wrist and hand the skin is normal, there is no swelling, erythema, or warmth. There is tenderness to palpation of the 1st dorsal compartment over the radial styloid. When asked to clinch her thumb in her fist and move the wrist into ulnar deviation, the pain over the radial styloid is reproduced. Neurovascular exam of the right upper extremity is normal. Radiographs of the right wrist and hand are unremarkable. | | |
| **Surgeon-written question** | **ChatGPT response** | **Surgeon responses** |
| What are the most likely diagnoses? | The most likely diagnosis for this patient is De Quervain's tenosynovitis. This condition is characterized by inflammation of the tendons and the synovial sheath within the first dorsal compartment at the radial styloid. It often results from repetitive hand and wrist movements, such as frequent texting or typing, which is consistent with the patient's history. The clinical presentation and positive Finkelstein's test (clinching the thumb in the fist and moving the wrist into ulnar deviation, reproducing pain over the radial styloid) are classic for De Quervain's tenosynovitis. Normal skin appearance, lack of swelling, erythema, warmth, and normal neurovascular exam also support the diagnosis. | The differential diagnosis is reasonable. However, there are some minor points that chat GPT had incorrect, including swelling at the first dorsal compartment associated with DeQuervains tenosynovitis^1^.  Additionally, the exam maneuver described as Finkelstein's test by having the participant place the thumb within the hand is, in fact, the Eichhoff test, which has been frequently misquoted as the Finkelsteins. Finkelstein's test is technically asking the patient to actively ulnar deviate the wrist before grasping the patient's thumb and passively flexing it into the palm^2^. |
| What is the workup for De Quervain’stenosynovitis? | The workup for De Quervain's tenosynovitis primarily relies on a thorough history and physical examination. In most cases, diagnostic imaging is not necessary as the diagnosis is primarily clinical. However, some steps in the workup may include:  1. History: A detailed history of the patient's symptoms, including onset, duration, aggravating and alleviating factors, and any history of repetitive hand or wrist movements or recent trauma.  2. Physical examination: Inspection of the wrist and hand for swelling, erythema, or warmth, as well as palpation for tenderness over the first dorsal compartment at the radial styloid. Assess the range of motion and strength of the wrist and thumb.  3. Finkelstein's test: Ask the patient to clench their thumb within their fist and then deviate the wrist towards the ulnar side. A positive test reproduces pain over the radial styloid, which is indicative of De Quervain's tenosynovitis.  4. Neurovascular examination: Evaluate the patient’s sensation, motor function, and muscular supply in the affected extremity to rule out other conditions or complications.  5. Imaging: Radiographs of the wrist and hand are typically not required for the diagnosis of De Quervain’s tenosynovitis. However, they may be attained if there is a suspicion of an alternative diagnosis, such as a fracture or arthritis. In some cases, ultrasound or MRI can be used to confirm the diagnosis or evaluate the severity of tendon inflammation, but these are usually reserved for more complicated cases or of conservative treatment fails.  6. Differential diagnosis: Consider other potential diagnosis such as intersection syndrome, wrist arthritis, or radial nerve entrapment, when the clinical presentation is not entirely consistent with De Quervain’s tenosynovitis. | The workup and differential are appropriate.  Physical examination may include induced pain with resisted thumb radial abduction. Please see the above answer for comment on Finkelstein’s test. |
| How would you treat De Quervain’stenosynovitis? | De Quervain's tenosynovitis can often be managed conservatively, especially in its early stages. The primary goal of treatment is to reduce inflammation and alleviate pain, allowing the patient to regain normal hand and wrist function. Treatment options include:  1. Activity modification: Advise the patient to avoid or minimize activities that cause pain and exacerbate the condition, such as repetitive thumb and wrist movements.  2. Rest: Encourage the patient to rest the affected hand and wrist to allow for healing.  3. Splinting: A thumb spica splint or brace can be used to immobilize the thumb and wrist, reducing irritation of the inflamed tendons.  4. Ice: Applying ice packs to the affected area for 15-20 minutes several times a day can help reduce inflammation and provide pain relief.  5. Non-steroidal anti-inflammatory drugs (NSAIDs): Over the counter medications like ibuprofen or naproxen can be used to reduce inflammation and provide pain relief.  6. Physical therapy: Strengthening and stretching exercises for the wrist and thumb, as well as techniques to improve the ergonomics of daily activities, can help prevent recurrence and promote healing  7. Corticosteroid injections: If conservative measures do not provide adequate relief, a corticosteroid injection into the affected tendon sheath may be considered to reduce inflammation and provide more rapid pain relief  8. Surgery: In cases where conservative treatments fail or the condition is severe, a surgical procedure called first dorsal compartment release may be considered. This involves releasing the tight sheath around the inflamed tenants to relieve pressure and allow for healing.  It is important to tailor the treatment plan to the individual patient, taking into accountthe severity of their symptoms, their occupation, and any other relevant factors. | The treatment plan is very reasonable and appropriate. |

1. Haddara MM, Kadar A, Ferreira LM, Suh N. Effect of a Flexor Digitorum Superficialis Hemitenodesis on Reducing Volar Plate Strains for Swan Neck Deformities. Hand N Y N. 2023;18(3):421-429. doi:10.1177/15589447211040877
2. Moore JS. De Quervain’s tenosynovitis. Stenosing tenosynovitis of the first dorsal compartment. J Occup Environ Med. 1997;39(10):990-1002. doi:10.1097/00043764-199710000-00011
