# Supplementary material for: Evaluation of a Large Language Model’s Ability to Assist in an Orthopedic Hand Clinic
Source: Hand (N Y). 2024 Jun 22;20(6):900–9. doi: 10.1177/15589447241257643 (PMC11571334; doi:10.1177/15589447241257643)
Supplement: sj-docx-5-han-10.1177_15589447241257643 – Supplemental material for Evaluation of a Large Language Model’s Ability to Assist in an Orthopedic Hand Clinic [file sj-docx-5-han-10.1177_15589447241257643.docx]

| **Supplemental Table 5 \| Swan Neck Deformity** | | |
| --- | --- | --- |
| A 65-year-old female presents with an 8-month history of snapping, locking, and deformity of her right long finger. Her past medical history is significant for rheumatoid arthritis and diabetes mellitus. She denies any injury to the finger and states that her symptoms began gradually. On physical examination of her right hand reveal no tenderness or erythema. At rest, the proximal interphalangeal joint is hyperextended and distal interphalangeal joint is flexed. Radiographs of the right hand reveal the ring finger deformity described above with no fractures or dislocations. | | |
| **Surgeon-written question** | **ChatGPT response** | **Surgeon responses** |
| What are the most likely diagnoses? | I am not a doctor, but based on the information provided, the most likely diagnoses for this 65-year-old female patient could be:  1. Trigger finger (stenosing tenosynovitis): This condition is characterized by a snapping or locking sensation in the finger, often due to inflammation of the tendon sheath. Rheumatoid arthritis and diabetes mellitus are known risk factors for developing trigger finger.  2. Swan neck deformity: This condition is characterized by hyperextension of the proximal interphalangeal (PIP) joint and flexion of the distal interphalangeal (DIP) joint, as described in the patient's presentation. Swan neck deformity is commonly associated with rheumatoid arthritis, which is part of the patient's medical history.  3. Boutonniere deformity: Although less likely based on the patient's symptoms, boutonniere deformity is another possibility to consider. It is characterized by flexion of the PIP joint and hyperextension of the DIP joint. Similar to swan neck deformity, boutonniere deformity is often associated with rheumatoid arthritis.  A thorough evaluation by a healthcare professional is necessary to determine the appropriate diagnosis and treatment for this patient. | Chat GPT has correctly identified swan neck deformity from the vignette. The differential is reasonable. There was a question of whether Boutonniere deformity is appropriate to include, given it is the opposite pathology as presented. |
| What is the workup for swan neck deformity? | I am not a doctor, but I can provide some general information on the workup for swan neck deformity. A healthcare professional would typically perform the following steps to diagnose and evaluate a swan neck deformity:  1. Medical history: The healthcare professional will take a detailed medical history, including information about any underlying conditions such as rheumatoid arthritis or other inflammatory disorders, previous injuries, and the onset and progression of symptoms.  2. Physical examination: A thorough physical examination of the affected hand and fingers will be performed, focusing on the range of motion, stability, and any deformities of the joints. The healthcare professional will also look for signs of inflammation, such as swelling, redness, or warmth, as well as any other abnormalities.  3. Radiographic imaging: X-rays of the affected hand can help to identify any joint damage, dislocations, or other structural abnormalities that may be contributing to this one activity. In some cases, more advanced imaging techniques such as MRI or ultrasound may be used to assess the soft tissue structures, including tendons and ligaments.  4. Laboratory tests: Blood tests may be ordered to evaluate the presence of inflammatory markers or to identify specific autoimmune conditions, such as rheumatoid arthritis, which could be contributing to the development of swan neck deformity.  Based on the findings from the medical history, physical examination, and any additional tests, healthcare professional will determine the severity of the swan neck deformity and recommend an appropriate course of treatment. Treatment options may include non-surgical interventions, such as splinting or occupational therapy, or surgical procedures to correct the deformity and restore function, depending on this underlying cause and severity of the condition. | Chat GPT accurately included a history of inflammatory diseases and trauma. However, the physical examination is very generic without mention of specific findings of swan neck deformity, including checking if there is a snapping swan neck deformity, if the patient can initiate flexion, seeing if the DIP flexion improves with passive flexion of the PIP joint, and checking for intrinsic tightness^1^. |
| What is the treatment for swan neck deformity? | I am not a doctor, but I can provide some general information on the treatment options for swan neck deformity. The treatment approach depends on the severity of the deformity, the underlying cause, and the patient's overall health and functional needs. Treatment options can be divided into non-surgical and surgical interventions.  Non-surgical treatments:  1. Splinting: Custom-made splints can be used to help maintain proper alignment of the finger joints, provide support, and alleviate pain. Splints are often used in the early stages of the deformity or for milder cases.  2. Occupational therapy: Occupational therapists can recommend exercises and techniques to help maintain or improve the range of motion, strength, and function of the affected fingers.  3. Anti-inflammatory medications: Over the counter or prescription non-steroidal anti-inflammatory drugs (NSAIDs) may be recommended to help reduce inflammation and alleviate pain.  4. Disease management: If the swan neck deformity is caused by an underlying condition such as rheumatoid arthritis, appropriate management of the condition with medications, lifestyle changes, or other interventions is essential to prevent further progression of the deformity.  Surgical treatments: If non-surgical treatments are ineffective or if the deformity is severe, surgical options may be considered.  1. Soft tissue procedures: These involve realignment or tightening of the tendons and ligaments around the affected joint to improve stability and correct the deformity.  2. Joint fusion (arthrodesis): This procedure involves fusing the affected joint to maintain proper alignment and provide stability. This can limit the range of motion, but it may be necessary in severe cases or if other treatments have not been successful.  3. Joint replacement (arthroplasty): In some cases, the damaged joint may be replaced with an artificial joint (prosthesis) to restore function and correct the deformity.  4. Bone removal or osteotomy: Removing a small portion of bone or reshaping the bone around the affected joint can help to correct the deformity and improve function.  The choice of treatment depends on the individual patients need, the severity of the deformity, and the underlying cause. A healthcare professional will evaluate the patient and recommend the most appropriate treatment plan. | The treatment plan is reasonable. However, it is very general. For example, splinting did not give examples of the types of splints used, such as a figure-8 splint. The surgical treatments were general rather than specific (for instance, they did not include examples of soft tissue procedures such as volar plate advancement or flexor digitorum superficialis hemitenodesis^2^. Although uncommon, osteotomies have been described^3^. |

1. Wu F, Rajpura A, Sandher D. Finkelstein’s Test Is Superior to Eichhoff’s Test in the Investigation of de Quervain’s Disease. J Hand Microsurg. 2018;10(2):116-118. doi:10.1055/s-0038-1626690
2. Lane R, Nallamothu SV. Swan-Neck Deformity. In: StatPearls. StatPearls Publishing; 2023. Accessed May 21, 2023. <http://www.ncbi.nlm.nih.gov/books/NBK525970/>
3. Moore JS. De Quervain’s tenosynovitis. Stenosing tenosynovitis of the first dorsal compartment. J Occup Environ Med. 1997;39(10):990-1002. doi:10.1097/00043764-199710000-00011
