# Supplementary material for: Evaluation of a Large Language Model’s Ability to Assist in an Orthopedic Hand Clinic
Source: Hand (N Y). 2024 Jun 22;20(6):900–9. doi: 10.1177/15589447241257643 (PMC11571334; doi:10.1177/15589447241257643)
Supplement: sj-docx-6-han-10.1177_15589447241257643 – Supplemental material for Evaluation of a Large Language Model’s Ability to Assist in an Orthopedic Hand Clinic [file sj-docx-6-han-10.1177_15589447241257643.docx]

| **Supplemental Table 6 \| Mallet Finger** | | |
| --- | --- | --- |
| A 35-year-old right hand dominant male presents with pain a deformity of his right long finger. His past medical history is unremarkable. The patient states that two days ago his long finger was “jammed” when attempting to catch a pass during a recreational basketball game. On physical examination there is mild swelling, tenderness to palpation, and deformity of his right long finger at the distal interphalangeal (DIP) joint. At rest the DIP joint is held in flexion and he is unable to actively extend the distal phalanx. Radiographs of the right long finger show no fractures or dislocations, but the long finger DIP joint is flexed approximately 45 degrees. | | |
| **Surgeon-written question** | **ChatGPT response** | **Surgeon responses** |
| What is the most likely diagnosis? | Based on the patient's history, physical examination, and radiographic findings, the most likely diagnosis is a mallet figure injury. A mallet finger, also known as baseball finger or drop finger, occurs when there is a disruption of the extensor tendon at the distal interphalangeal (DIP) joint, resulting in the inability to actively extend the distal phalanx. This type of injury is commonly caused by sudden force applied to the tip of a finger when it is in a flexed position, such as during sports like basketball. The characteristic deformities is a flexed DIP joint, and it can occur with or without an associated avulsion fracture. | Chat GPT was able to come to the correct diagnosis of mallet finger. It did not provide a more extensive differential diagnosis. |
| What is the workup for mallet finger injury? | The workup for a mallet finger injury typically involves the following steps:  1. History: Obtain a thorough history of the injury, including mechanism, onset, and any prior similar injuries.  2. Physical examination: Assess for pain, swelling, tenderness, and deformity of the affected finger, specifically at the distal interphalangeal (DIP) joint. Evaluate the patient's ability to actively extend the distal failings.  3. Radiographs: Obtain radiographs of the affected finger, including anteroposterior (AP) lateral, and oblique views. This is to assess for any associated fractures or dislocations and to evaluate the degree of flexion at the DIP joint.  4. Additional imaging: In some cases, more advanced imaging like ultrasound or magnetic resonance imaging (MRI) may be necessary to further assess the soft tissues and tendon involvement, particularly if there is suspicion of a more complex injury.  After the workup is completed, an appropriate treatment plan can be formulated based on the severity of the injury and any associated injuries. The mainstay of treatment for most mallet finger injuries is conservative management with splinting, but surgery may be indicated in some cases. | The workup for mallet finger is appropriate and thorough |
| What is the treatment for mallet finger injury? | The treatment for a mallet finger injury depends on the severity of the injury, presence of associated fractures, and patient factors. The mainstay of treatment for most mallet finger injuries is conservative management with splinting. Here are the general treatment options:  1. Conservative management with splinting: For most cases especially when there is no associated fracture or the fracture involves less than 1/3 of the joint surface, a mallet finger splint is used. The affected finger is immobilized in a splint that keeps the distal interphalangeal (DIP) joint in slight hyperextension. The splint should be worn continuously for 6-8 weeks, and the patient should avoid flexing the DIP joint during this. After this period, the splint can be gradually weaned off, and the patient can start range of motion exercises. It's important to monitor for complications, such as skin breakdown, infection, or joint stiffness, during the splinting period.  2. Surgical intervention: Surgery may be indicated in cases with associated fractures involving more than 1/3 of the joint surface, or when there are significant joint subluxation or displacement. Different surgical techniques can be used, such as closed reduction and percutaneous pinning, open reduction and internal fixation, or tendon repair using suture anchors. Surgical intervention carries risk, such as infection, nerve injury, or stiffness, and should be considered on a case-by-case basis.  3. Post treatment rehabilitation: After the splinting period or surgery, a gradual return to activity and rehabilitation is important. A hand therapist may be involved to help guide the patient through range of motion exercises, strengthening, and functional activities to help restore normal function. It is crucial to discuss treatment options with the patient and consider their individual needs, expectations, and occupation when formulating the treatment plan. | Chat GPT included a concise and appropriate treatment plan with potential surgical management indications. |
